# GPER-independent inhibition of adrenocortical cancer growth by G-1 involves ROS/Egr-1/BAX pathway

**DOI:** 10.18632/oncotarget.23314

**Published:** 2017-12-14

**Authors:** Ivan Casaburi, Paola Avena, Arianna De Luca, Rosa Sirianni, Vittoria Rago, Adele Chimento, Francesca Trotta, Carmela Campana, William E. Rainey, Vincenzo Pezzi

**Affiliations:** ^1^ Department of Pharmacy, Health and Nutritional Sciences, University of Calabria, Arcavacata di Rende, Cosenza, Italy; ^2^ Departments of Molecular & Integrative Physiology and Internal Medicine, University of Michigan, Medical School, Ann Arbor, MI, USA

**Keywords:** GPER agonist G-1, Egr-1, ROS, mitochondrial apoptotic pathway, adrenocortical cancer cell

## Abstract

We previously demonstrated that treatment of the H295R adrenocortical cancer cell line with the non-steroidal, high-affinity GPER (G protein-coupled estrogen receptor 1) agonist G-1 reduced tumor growth *in vitro* and *in vivo* through a GPER independent action. Moreover, we observed that G-1 treatment induces cell-cycle arrest and apoptosis following a sustained ERK1/2 activation. However, the precise mechanisms causing these effects were not clarified. Starting from our preliminary published results, we performed a microarray study that clearly evidenced a strong and significative up-regulation of EGR-1 gene in H295R cells treated for 24h with micromolar concentration of G-1. The microarray findings were confirmed by RT-PCR and Western-blot analysis as well as by immunofluorescence that revealed a strong nuclear staining for EGR-1 after G-1 treatment. EGR-1 is a point of convergence of many intracellular signaling cascades that control tumor cell growth and proliferation as well as others that relate to cell death machinery. Here we found that the increased Egr-1 expression was a consequence of G-1-mediated ROS-dependent ERK activation that were promptly reversed by the presence of the antioxidant n-acetyl-cysteine. Finally, we observed that silencing EGR-1 gene expression reversed the main effects induced by G-1 in ACC cells, including upregulation of the negative regulator of cell cycle, p21^Waf1/Cip1^ and the positive regulator of mitochondrial apoptotic pathway, BAX, as well as the cell growth inhibition. The identified ROS/MAPK/Egr-1/BAX pathway as a potential off-target effect of the G-1 could be useful in implementing the pharmacological approach for ACC therapy.

## INTRODUCTION

Rarity, complex pathogenesis and limited therapeutic options are the main features to deal with when addressing adrenocortical cancer (ACC). Mitotane is the drug that is currently used for the treatment of advanced and metastatic ACC [1]. However, toxicity, narrow therapeutic window and unwanted side effects represent major limitations to its use as well as therapeutic success [2, 3]. Thus, more effective and specific treatment options are needed.

The majority of currently published studies that investigate the cause of ACC, has analyzed only single pathways of signal transduction, but it is becoming clear that ACC pathogenesis involves integration of signals and the interplay of downstream pathways [2]. Among these, the IGF system and estrogen-dependent pathways appear to be of particular interest. In fact, IGFII is overexpressed in 90% of ACC and its effects are mediated through its receptor, IGF1R, resulting in the activation of kinase-dependent pathways [2]. Recently, we demonstrated a pivotal role of estrogen receptor alpha (ERα) in the activation of these same IGF pathways in response to estrogens [4]. Accordingly, in ACCs we found that ERα expression is up-regulated and estradiol enhances proliferation of the H295R adrenocortical cancer cells [5, 6]. Moreover, tamoxifen, a selective estrogen receptor modulator (SERM), inhibits estrogen- and IGF-II-stimulated H295R adrenocortical cancer cell proliferation *in vitro* and reduces H295R xenografts growth [4]. However, in addition to ERα modulation, it has been demonstrated that tamoxifen can act as full agonist on the G protein-coupled estrogen receptor (GPER) [7].

G-1 (1-[4-(6-bromobenzo [1, 3]dioxol-5yl)-3a,4,5,9b-tetrahydro-3H-cyclopenta-[c]quinolin-8-yl]-ethanone), a non-steroidal GPER agonist, has been developed to dissect GPER-mediated estrogen responses from those mediated by classic estrogen receptors, ERα and β [8]. Since its discovery, G-1 has been used in a large number of studies to investigate the role of GPER in numerous systems including the nervous, immune, reproductive and vascular systems as well as cancer [9–11]. It is worth mentioning that the biological activities triggered by G-1-mediated GPER activation, such as cell proliferation [7, 12] and/or cell death [10, 13], appear to be cell type specific and dependent on the ERs expression pattern [14]. The picture becomes even more complex considering the effects elicited by G-1 in a GPER-independent manner [15]. According to our previous study, G-1 is able to inhibit ACC cell growth both *in vitro* and *in vivo* [16]. In particular, cell cycle arrest and activation of the intrinsic apoptotic pathway were triggered by G-1 via long-term sustained ERK phosphorylation in a GPER-independent fashion. The aim of this study was to define in detail the G-1-activated pathways in adrenocortical cancer and associated with cell death in response to G-1 treatment. Transcription analysis defined the gene expression alternations in H295R cells exposed to G-1, that were further investigated.

## RESULTS

### G-1-inducible genes in H295R cells defined by microarray analysis

**Figure 1:**
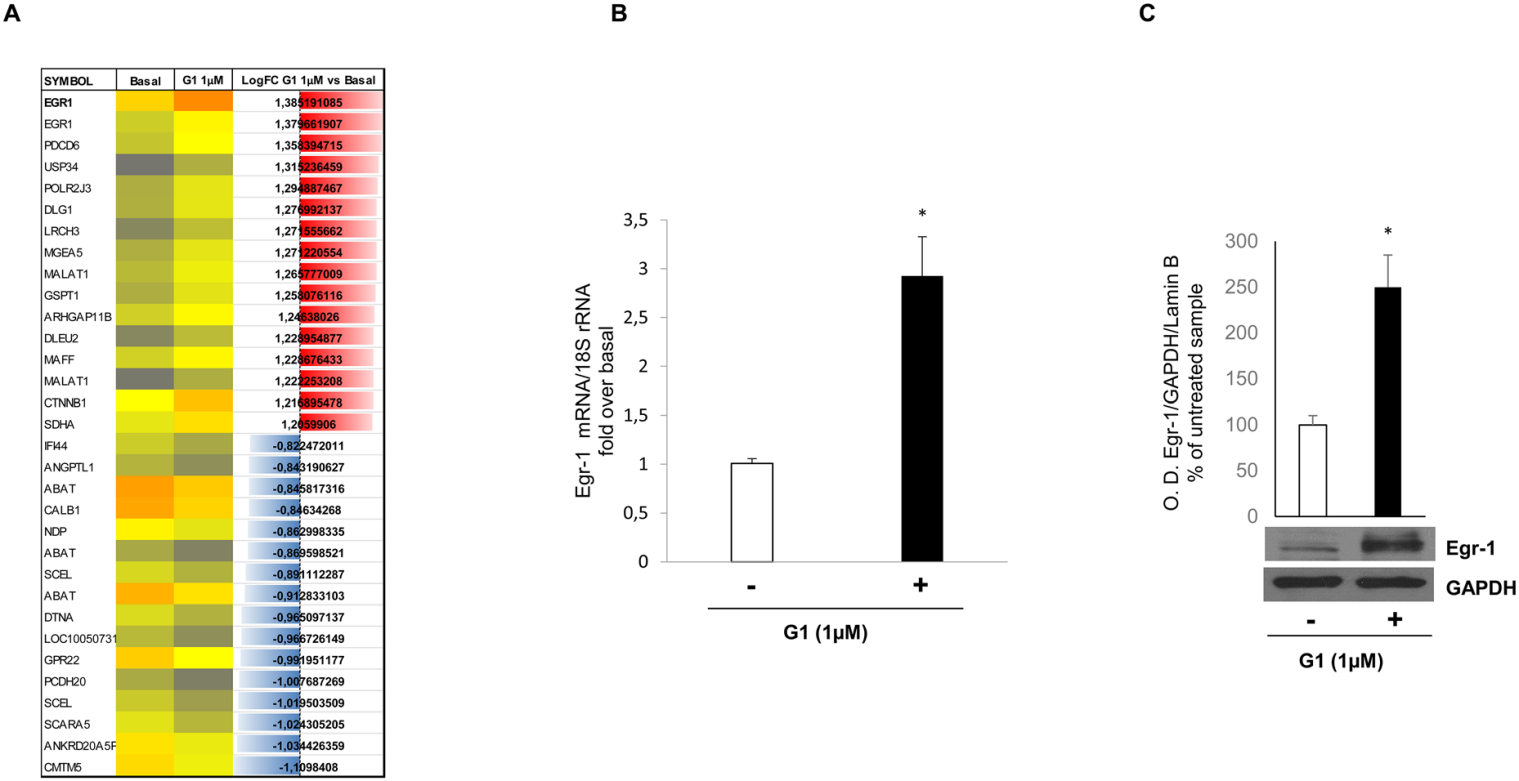
G-1 stimulation induces Egr-1 expression in H295R cells **(A)** representative microarray analysis with the most highly up-regulated (blu) and down-regulated (red) genes. **(B)** Egr-1 mRNA expression in H295R cells treated for 24 h with vehicle (–) or G-1 (1μM) was analyzed by real time RT-PCR. Each sample was normalized to 18S rRNA content. Final results are expressed as n-fold differences of gene expression relative to calibrator. Data represent the mean ± SD of values from at least three separate RNA samples (^*^p < 0.001, versus calibrator). **(C)** Total extracts from H295R cells left untreated (–) or treated with G-1 (1μM) for 24 h were resolved by SDS-PAGE and subjected to immunoblot analysis using specific antibodies against human Egr-1. Blots are representative of three independent experiments with similar results. GAPDH served as loading controls. The upper graph are optical densities (O. D.) ±SD, ^**^p < 0.01.

To identify genes that were induced by G-1 in H295R cells, we cultured cells with or without G-1 (1μM) for 24h. This exposure time point was selected from previous results demonstrating 24h as the first visible signs of G-1-induced apoptosis. Total RNA was extracted and subjected to microarray analysis using Affymetrix human U133 plus 2.0 GeneChips. By using the GeneChips analysis suite, we sorted genes that were either up- or down-regulated by greater than two-fold following G-1 treatment in three independent experiments (Figure 1A). Several genes were modulated by G-1 in all three experiments but we focused our attention on Egr-1, a gene with a role in both cell growth and apoptosis [17]. Egr-1 was up-regulated by 2.9-fold, a result that was further confirmed in H295R cells at both transcriptional and post-transcriptional level (Figure 1B) by real-time reverse transcription PCR (qRT-PCR) and Western blot analyses, respectively.

### G-1 induces Egr-1 nuclear translocation in H295R cells

**Figure 2:**
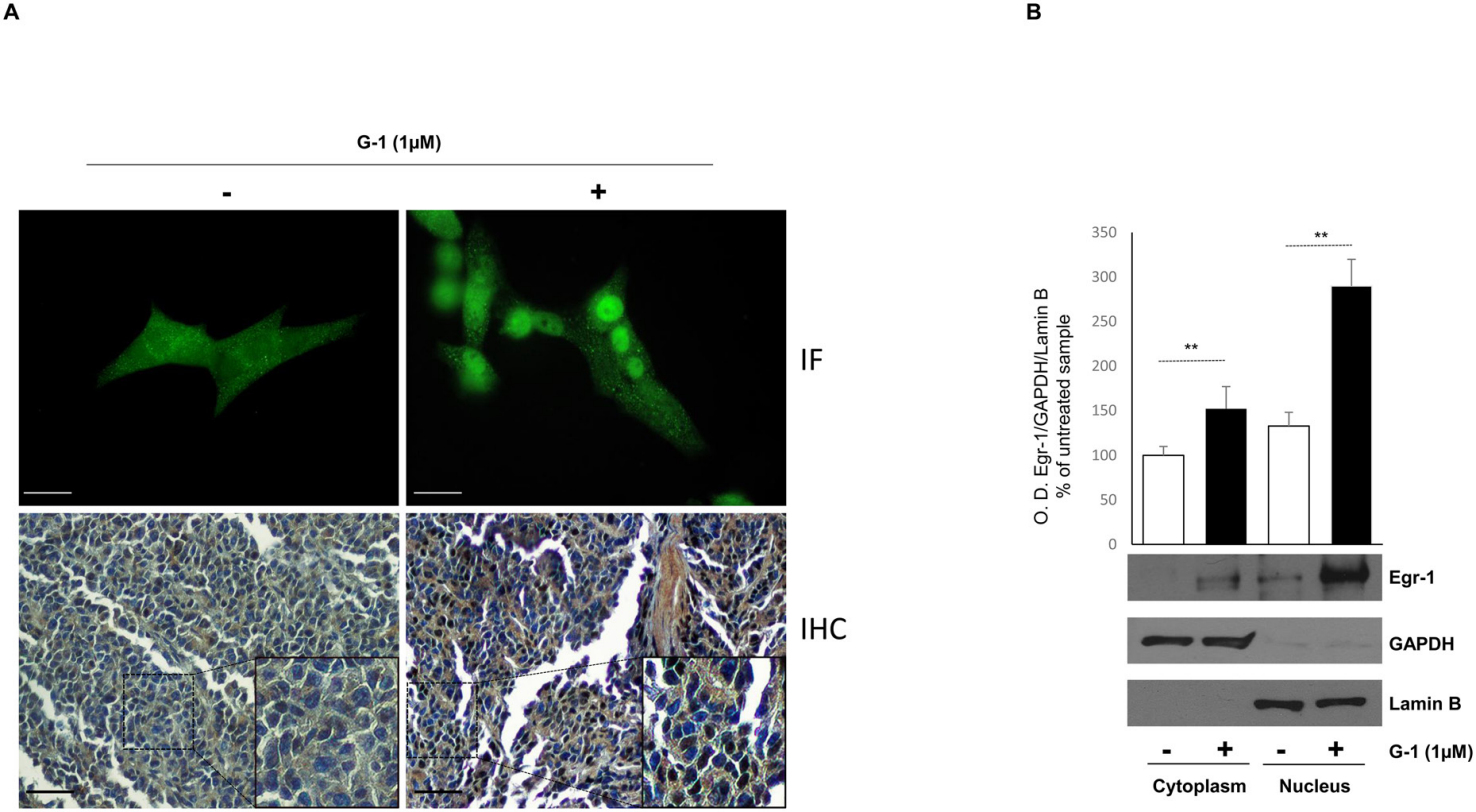
G-1 induces nuclear translocation of Egr-1 in H295R cells **(A)** (Upper panel) positive nuclear fluorescent staining for Egr-1 expression in H295R cells treated for 24 with vehicle (−) or G-1 (1μM). (A) (Lower panel) immunohistochemical staining for Egr-1 in untreated and G-1 treated H295R xenograft tumors. Insets are an higher magnification (400x) of the marked area. **(B)** Cytoplasmic and nuclear extracts from H295R cells left untreated (–) or treated with G-1 (1μM) for 24 h were resolved by SDS-PAGE and subjected to immunoblot analysis using specific antibodies against human Egr-1. Blots are representative of three independent experiments with similar results. GAPDH and Lamin B served as loading controls. The upper graph are optical densities obtained from three independent experiments ±SD, ^**^p < 0.001.

Egr-1 is a nuclear transcription factor that represents a point of convergence of many intracellular signaling pathways [18]. To verify its nuclear translocation upon G-1 treatment we used different experimental approaches. First, we performed immunofluorescence assay where untreated or G-1-treated cells were fixed and incubated with anti-Egr-1 antibody followed by an incubation with a secondary FITC-conjugated antibody. In Figure 2A (upper panel) positive nuclear staining for Egr-1 is clearly visible after 24h treatment with G-1 while in untreated control cells Egr-1 appears as dotted areas within the cytoplasm and around the nucleus. Moreover, immunohistochemistry using tissue slides of H295R xenograft tumors derived from mice treated with vehicle and G-1 showed an increased cytoplasmic and nuclear staining for Egr-1 after G-1 exposure (Figure 2A, lower panel), as demonstrated by Allred immunostaining score (Table 1) [16]. In addition, Western blot analysis of cytoplasmic and nuclear protein clearly showed that Egr-1 accumulates within the nuclei after 24h treatment with G-1 1μM (Figure 2B). These results clearly showed that G-1 causes Egr-1 activation in H295R cells.

**Table 1:** Egr-1 immunoreactivity (Allred score) in xenografted H295R cells

	Untreated control cells	G-1 1μM
Cytoplasm	1	3^*^
Nucleus	3	7^*^

Total immunostaining score (n: 6-7 serial section for each treatment): Proportion score + Intensity score (range 0–8). Significant difference: ^*^p < 0.05 compared to untreated control cells.

### G-1 induces ROS-dependent Egr-1 upregulation

**Figure 3:**
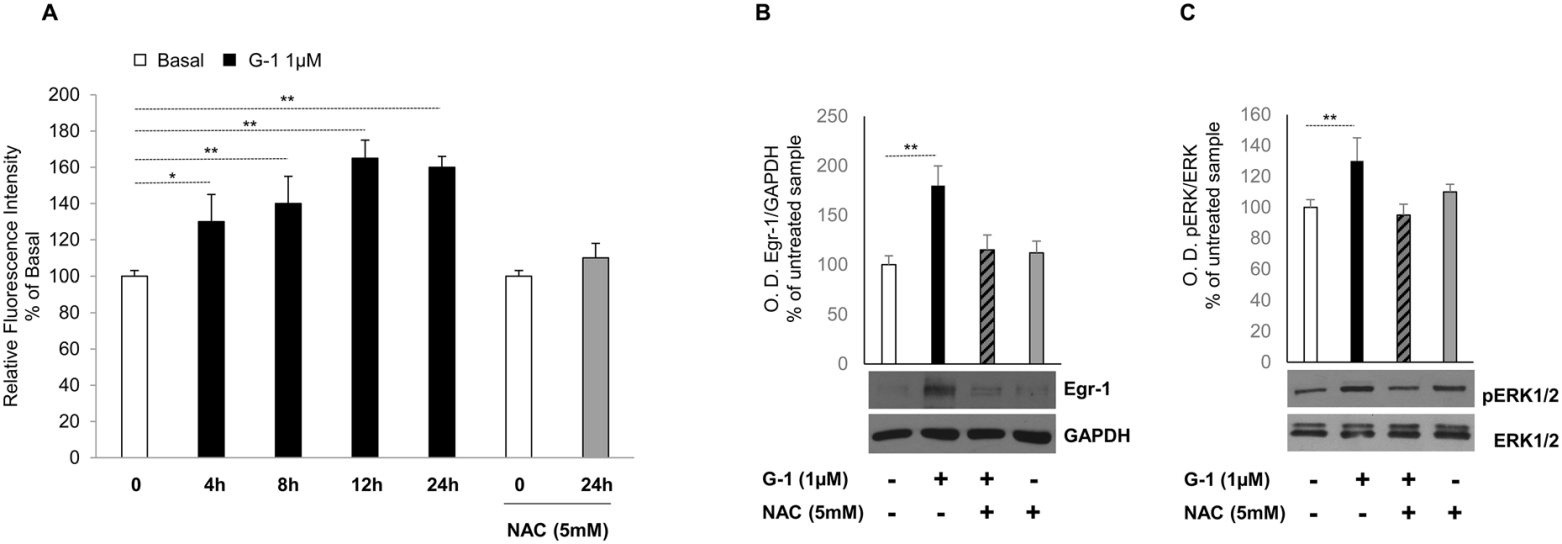
G-1-induced Egr-1 expression is associated with ROS generation in H295R cells **(A)** H295R cells were treated for different times with G-1 (1μM) and then incubated with 5-(and-6)-chloromethyl-2′,7′-dichlorodihydrofluorescein diacetate (CM-H2DCFDA). Where indicated cells were pre-treated for 1 h with NAC (5mM), and then treated with G-1 (1μM) for 24 h and finally incubated with 5-(and-6)-chloromethyl-2′,7′-dichlorodihydrofluorescein diacetate (CM-H2DCFDA). Relative fluorescence intensity was monitored using a microplate reader (Ex/Em: ∼492–495/517–527 nm). ROS generation was expressed as relative fluorescence intensity of treated cells versus untreated control cells. Each column represents the mean ± SD of three independent experiments. **(B** and **C)** H295R cells were pre-treated with NAC (5 mM) for 1h and then treated for 24h with vehicle (−) or G-1 (1μM). Western blot analysis of Egr-1 and GAPDH, used as a loading control (B), or pERK1/2 and total ERK1/2 (C) was performed on equal amounts of total proteins. Blots are representative of three independent experiments with similar results, (^*^p < 0.05, ^**^p < 0.001) compared to untreated control sample.

Several studies indicate that Egr-1 is induced by a number of extracellular stimuli, including growth factors, mitogens, cytokines and injury-related stimuli as well as many inducers of ROS-mediated signaling and inflammation leading to cell death [18]. In our previous work we demonstrated that G-1 inhibits H295R cell growth by activating the mitochondrial apoptotic pathway [16] and one of the mechanisms able to induce mitochondria-dependent apoptosis is through the generation of ROS. Therefore, we investigated the ability of G-1 to generate intracellular ROS. To this aim, H295R cells were treated for different times with G-1 and then incubated with CM-H2DCFDA. H2DCFDA is rapidly taken up by the cells where, is converted into non-fluorescent CM-H2DCF by esterase action and subsequently oxidized by intracellular oxidants, such as ROS, into highly fluorescent CM-DCF. The fluorescence intensity was monitored using a microplate reader (Ex/Em: ∼492–495/517–527 nm). Results illustrated in Figure 3A show how G-1 is able to increase ROS production up to 24h, an event that was promptly reversed when cells were pre-treated for 1h with 5mM NAC, a commonly used reactive oxygen intermediate scavenger. Because G-1 generated ROS and Egr-1 expression were maximal between 12 and 24h, (data not shown), potential ROS-induced regulation of Egr-1 expression was investigated. Immunoblotting data indicated that blocking the generation of ROS by pre-treating cells with NAC markedly prevented G-1-induced Egr-1 protein expression (Figure 3B). In addition, we already demonstrated that G-1 treatment caused sustained ERK1/2 phosphorylation leading to cell death by apoptosis [16]. Here we also showed that pre-treatment of H295R cells with NAC prevented G-1-induced ERK1/2 phosphorylation (Figure 3C). These results clearly show the requirement of ROS formation in ERK1/2 activation by G-1.

### G-1 activates Egr-1/BAX signaling in H295R cells through ERK signaling

**Figure 4:**
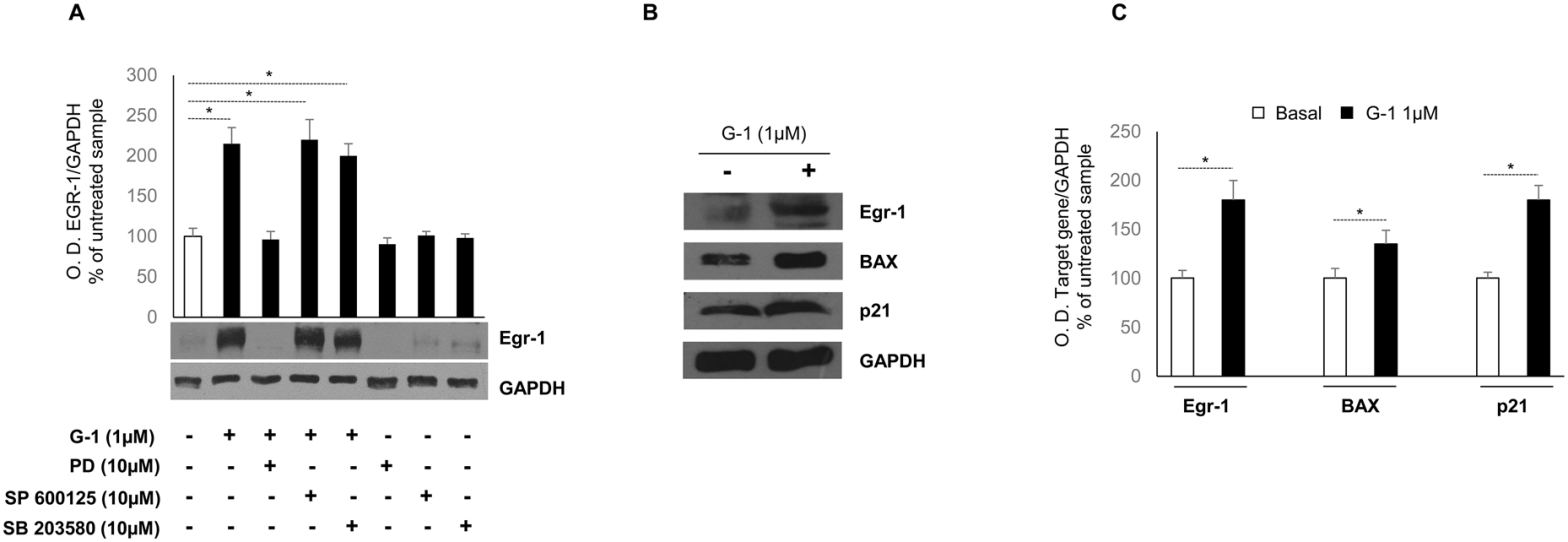
Role of MAPKs and Egr-1/BAX signaling in G-1-treated H295R cells **(A)** H295R cells were treated for 24 h with vehicle (−) or G-1 (1μM) alone or combined with PD98059 (10μM), SP600125 (10μM), SB203580 (10μM). Western blot analysis of Egr-1was performed on equal amounts of total proteins. GAPDH was used as a loading control. Blots are representative of three independent experiments with similar results. Graph represents mean of Egr-1 optical density (O.D.) from three independent experiments with similar results normalized to GAPDH content (^*^p < 0.01 compared to untreated control sample assumed as 100). **(B)** Total proteins from H295R cells left untreated (–) or treated with G-1 (1μM) for 24 h were resolved by SDS-PAGE and subjected to immunoblot analysis using specific antibodies against human Egr-1, BAX, p21^Waf1/Cip1^. Blots are representative of three independent experiments with similar results. GAPDH served as loading control. **(C)** Histograms represent the mean ± SD of band intensities evaluated as optical density (O.D.) arbitrary units and expressed as the percentage of the control assumed as 100%, ^*^p < 0.01 compared with untreated cells.

The existence of a close association between ROS formation and the activation of MAPK signaling has long been known [19, 20]. Indeed, in mammalian cells there are three well-defined subgroups of MAPKs: the extracellular signal regulated kinases (ERKs), the c-Jun N-terminal kinases (JNK), and the p38-MAPK. The three subgroups of MAPKs are involved in both cell growth and cell death, and the tight regulation of these pathways is paramount in determining cell fate [21]. To determine whether the activation of ERK, JNK and p38-MAPK participated in G-1-mediated upregulation of Egr-1 expression, the effects of specific inhibitors for ERK (PD98059), JNK (SP600125), and P38 (SB203580) were tested on G-1-treated cells (Figure 4A). The results showed that pretreatment with PD98059 did abrogate G-1-induced Egr-1 expression. By contrast, pharmacological inhibition of p38-MAPK or JNK activity failed to suppress G-1-induced Egr-1 expression. Moreover, upregulation of Egr-1 expression was concomitant with an increased expression, at both transcriptional (data not shown) and post-transcriptional (Figure 4B and 4C) levels, of two known Egr-1 target genes, specifically p21Waf1/Cip1 and BAX. Both proteins have been shown to play a role in G-1-mediated H295R cell cycle arrest and apoptosis [16].

### Egr-1 gene silencing abolishes G-1-mediated effects on H295R cells

**Figure 5:**
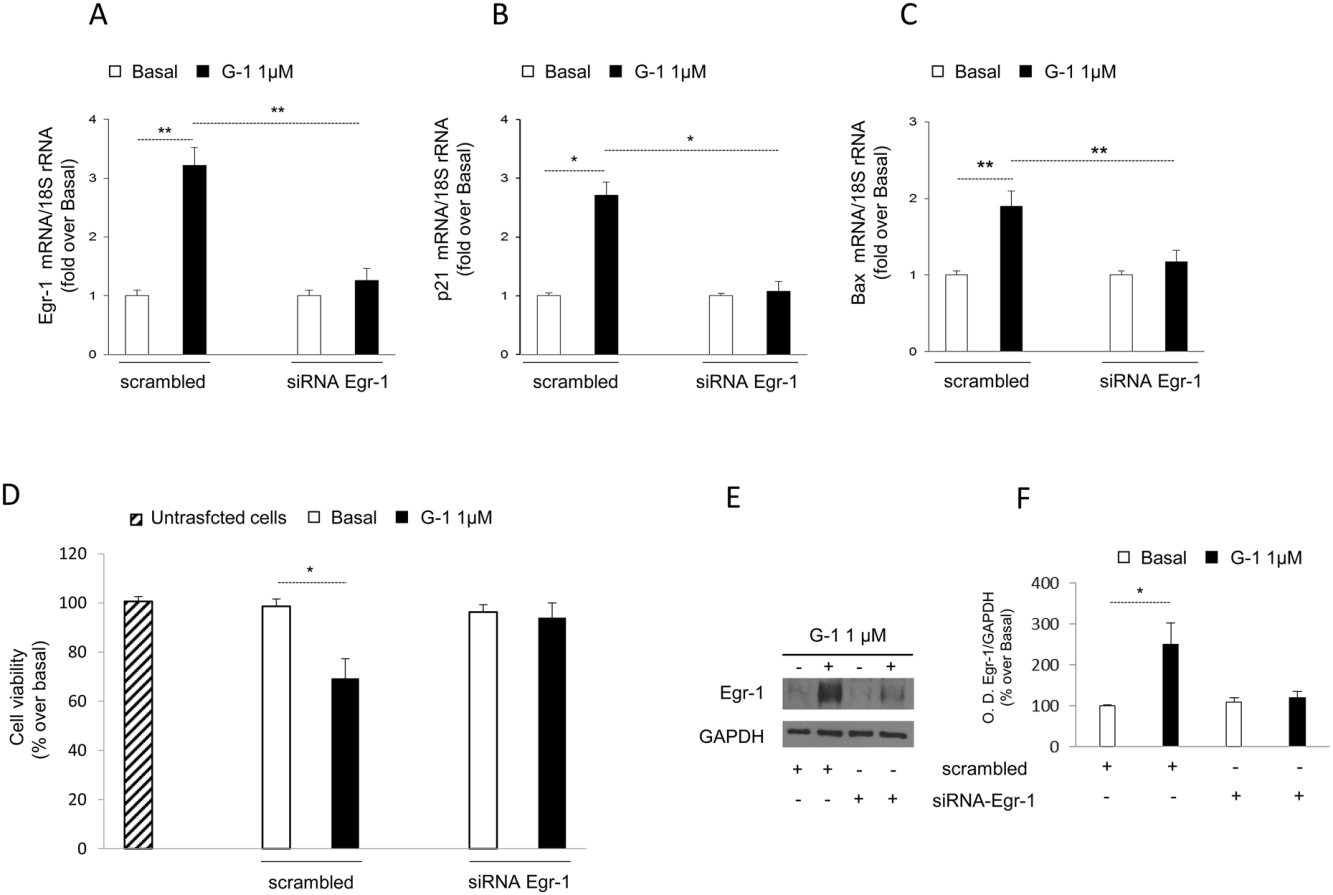
Egr-1 gene silencing reversed G-1-induced effects on H295R **(A-C)** Q-PCR analysis was performed in H295R cells to evaluate the expression of Egr-1 or p21^Waf1/Cip1^ or BAX mRNA in the absence (-) or presence of control siRNA (scramble) or siRNA specific for Egr-1. Ribosomal 18S subunit mRNA was used as internal control. **(D)** MTT assay was performed on H295R cells untransfected, transfected with specific siRNA for Egr-1 or scrambled siRNA for 24h, as indicated in Materials and Methods, and then left untreated (-) or treated with G-1 for additional 24h. **(E)** To assess reduced Egr-1 protein expression in cells used for viability assay, random wells from untreated or G-1 treated cells were used for protein extraction and Western blotting analysis. GAPDH protein expression was used as loading control. (F) Histograms represent the mean ± SD of band intensities evaluated as optical density (O.D.) arbitrary units and expressed as the percentage of the control assumed as 100%. Data represent the mean ± SD of three separate experiments each in triplicate. ^*^p < 0.05, ^**^p < 0.001, compared with untreated cells.

To further define the prominent role of Egr-1 in G-1-mediated effects, we decided to silence Egr-1 gene expression. In this experimental condition, we first examined the effect of gene silencing on the ability of G-1 to upregulate Egr-1 target genes such as p21^Waf1/Cip1^ and BAX. As show in Figure 5, silencing of Egr-1 gene expression (Figure 5A) abrogated the transcription of both p21^Waf1/Cip1^ (Figure 5B) and BAX (Figure 5C) genes following G-1 treatment. Considering that these two genes are responsible for the inhibitory effects exerted by G-1 on H295R cell growth [16], we also investigated the impact of Egr-1 gene silencing on G-1-mediated inhibition of cell viability. Results showed in Figure 5D clearly demonstrated that G-1 was unable to reduce the viability in cells with an impaired expression of Egr-1 (Figure 5E and 5F).

## DISCUSSION

In a previous study we showed that micromolar concentrations of G-1 significantly suppressed H295R cell proliferation both *in vitro* and *in vivo* by the activation of the intrinsic mitochondria-mediated apoptotic pathway and the associated molecular mechanism involving the long and sustained phosphorylation of ERK1/2 [16].

Since G-1 has been reported to be a selective GPER agonist, we expected that knockdown of GPER in H295R cells, might completely abrogate H295R cell growth inhibition. Surprisingly, when we effectively knocked down GPER expression by siRNA, we found that the inhibitory effects of G-1 were only partially reversed. This finding suggested that G-1 might also suppress H295R cell proliferation in a GPER-independent manner. This hypothesis was supported by others describing the inhibitory role of G-1 regardless of GPER expression in both breast and ovarian cancer cell lines [15], as well as in human ovarian endometriosis stromal [22] and vascular smooth muscle cells [23].

The aim of the this study was to clarify the potential intracellular targets and the mechanisms activated by G-1 to inhibit ACC cell proliferation and induce apoptosis. For these reasons, we started our study with a microarray analysis of H295R cells treated with G-1. Of several genes that were either up-regulated or down-regulated by G-1 treatment, we focused our attention on upregulated Egr-1 gene for two main reasons. First, Egr-1 is involved in G-1 induced signaling in different tumor cells [24]; second, Egr-1 has a dichotomic function since it can work as oncogene but also as tumor suppressor [18].

Egr-1, also known as NGFI-A, Zif268, T1S8 and krox-24, is a Cys2-His2-type zinc-finger transcription factor, is a member of the immediate-early gene family mapping to chromosome 5 [25]. Its modular structure ensures a rapid response to different stimuli which results in the transcription of several target genes involved not only in the regulation of cell growth and differentiation, but also in determining apoptosis [18, 26].

Our results clearly showed that G-1 significantly upregulated Egr-1 expression at both the transcript and protein level. Moreover, its nuclear translocation, as evidenced by immunofluorescence and immunohistochemistry assays, highlighted G-1 ability to increase and activate Egr-1.

In addition to its regulation by multiple extracellular stimuli [18], Egr-1 can be considered a redox-regulated gene because it is activated by all inducers of ROS-mediated signaling and inflammation [27–31]. This feature is due to the presence of oxidative stress-responsive DNA sequence within its promoter region [32], and most inducers of ROS-mediated signaling pathways increase the levels of Egr-1 [27–31]. Here we determined that G-1 induces ROS production in a GPER-independent manner. It is worth noting that other tumor types respond to G-1 treatment activating ROS production [33–35]. However, in contrast to our observation on a GPER-independent mechanism, those studies claim the involvement of G-protein receptor. Indeed, we previously demonstrated that doses of G-1 1μM and onward elicit GPER-independent effects as supported by RNA interference experiments [16]. In agreement with previous observations on the biological effects exerted by G-1, we proved that G-1 activated the intrinsic apoptotic pathway where mitochondria play a key role as target of different stress stimuli including, above all, ROS [36]. Therefore, we demonstrated that treatment of ACC cells with G-1 results in a significant increase in ROS production that was no longer detectable in the presence of the antioxidant NAC. The same observation was recently reported for colon cancer [37]. Taking into account the critical role of Egr-1 in coordinating cellular events following oxidative stress [38–40], we showed that the presence of NAC was also able to reverse G-1-mediated up-regulation of Egr-1 expression. The scavenger effect of NAC on the G-1-dependent ROS increase has also been shown with regard to the activation of ERK1/2, one of the major ROS targets that is responsible for the up-regulation of the pro-apoptotic factor BAX in ACC cells [16]. Other important targets of ROS are represented by JNK and p38MAPK. Therefore, using specific inhibitors of these pathways, the results clearly demonstrated a selective involvement of ERK pathway in mediating the G-1 induced Egr-1 expression. By contrast, no role was observed for both JNK and p38. These findings are in agreement with those of others revealing that MAPKs regulate the expression of Egr-1 under stress conditions and elevated ROS levels [41, 42].

Finally, after silencing Egr-1 we confirmed its involvement in cell cycle arrest and mitochondrial apoptotic process. In fact, the upregulation of p21^Waf1/Cip1^ and BAX expression seen under G-1 treatment, and consequently, the inhibitory effect on cell viability, were promptly reversed by silencing Egr-1 gene expression. These last results underlie the prominent role of Egr-1 in the inhibitory effects exerted by G-1 on ACC cells.

In conclusion, despite several reports indicate G-1 ability to increase ROS production and then cell apoptosis, a clear mechanism has not been defined. We can only confirm what we have already reported [16] that mitochondrial dysfunctions and mitochondrial-mediated apoptotic pathways are involved in the inhibitory effects of G-1.

Extending the findings of our previous study [16] we demonstrate that 1) G-1 increases intracellular ROS production in ACC cells, 2) ROS increases Egr-1 mRNA and protein expression most probably trough the activation of ERK1/2 signaling, 3) the increased expression and activation of Egr-1 causes a marked up-regulation of its target genes such as the pro-apoptotic factor BAX and the cell cycle inhibitor p21^Waf1/Cip1^ responsible for the inhibitory effect exerted by G-1 on ACC cells. The identification of ROS/MAPK/Egr-1/BAX pathway as specific target activated by micromolar concentration of G-1 gives indication for new pharmacological approaches addressed to ACC therapy.

## MATERIALS AND METHODS

### Cell culture and MTT assay

H295R cells were cultured as previously described [16]. Cell monolayers were subcultured onto 60 mm dishes for protein and RNA extraction (4 × 10^6^ cells/plate) and 12 well/plate (1 × 10^5^ cells per well) for the MTT assay. Prior to experiments, cells were starved overnight in DMEM/F-12 medium without phenol red. Cells were treated with (G-1, 1μM) (Tocris Bioscience, Bristol, UK) in DMEM/F-12 containing 2, 5% FBS-DCC (fetal bovine serum dextran-coated charcoal-treated). Inhibitors such as PD98059 (10μM), SB203580 (10μM), SP600125 (10 μM) (Calbiochem, Merck KGaA, Darmstadt, Germany) and ROS scavenge molecule NAC (N-acetyl cysteine, Sigma) (5mM) were used 1h prior to G-1. Cell viability was measured using MTT assay (Sigma-Aldrich) as already reported [43]. Each experiment was performed in triplicate and the optical density was measured at 570 nm in a spectrophotometer. Experiments were repeated three times.

### Microarray

RNA from H295R cells untreated (basal) or treated for 24 hour with G-1 (1μM) were hybridized to an Affymetrix human HG-U133plus oligonucleotide two-microarray set containing more than 54,000 probe sets representing over 38,500 independent human genes (Affymetrix, Santa Clara, CA). The arrays were scanned at high resolution using an Affymetrix GeneChip Scanner 3000. Results were analyzed using GeneSpring version 6.1 software (Silicon Genetics, Redwood City, CA). Pure signal values were normalized using a list of 100 normalization control probe sets published by Affymetrix and used to identify genotypic differences between untreated and treated cells. Probe ID for Egr-1: 227404 PM.

### RNA extraction, reverse transcription and real time PCR

TRizol RNA isolation system (Invitrogen, Carlsbad, CA, USA) was used to extract total RNA from H295R. Each RNA sample was treated with DNase I (Invitrogen), and purity and integrity of the RNA were confirmed spectroscopically and by gel electrophoresis before use. One microgram of total RNA was reverse transcribed in a final volume of 30 μl using the ImProm-II Reverse transcription system kit (Promega Italia S.r.l., Milano, Italy); cDNA was diluted 1:2 in nuclease-free water, aliquoted, and stored at −20°C. The nucleotide sequences for Egr-1, p21^Waf1/Cip1^ and BAX amplification were: Egr-1, forward, 5′-CTCTCCAGCCTGCTCGTC-3′, and reverse, 5′-AGCAGCATCATCTCCTCCAG-3′; p21^Waf1/Cip1^, forward, 5‘-CATGACAGATTTCTACCACTCC-3′ and reverse, 5‘-AAGATGTAGAGCGGGCCTTT-3′; BAX, forward 5‘-GCTCTGAGCAGATCATGAAGACA-3′ and reverse 5‘-TCGCCCTGCTCGATCCT-3′. The nucleotide sequences for 18S amplification were forward, 5′-CGGCGACGACCCATTCGAAC-3′, and reverse, 5′-GAATCGAACCCTGATTCCCCGTC-3′. PCR reactions were performed in the iCycler iQ Detection System (Bio-Rad Laboratories S.r.l., Milano, Italia) using 0.1 μmol/L of each primer, in a total volume of 30 μl reaction mixture following the manufacturer’s recommendations. SYBR Green Universal PCR Master Mix (Bio-Rad) with the dissociation protocol was used for gene amplification; negative controls contained water instead of first-strand cDNA. Each sample was normalized to its GAPDH content. The relative gene expression levels were normalized to a calibrator (Basal, untreated H295R cells). Final results were expressed as n-fold differences in gene expression relative to GAPDH and calibrator, calculated using the ΔΔCt method as previously published [16].

### Egr-1 gene silencing

Cells were plated with regular growth medium two days before transfection to 50–60% confluence.

The day of transfection the medium was changed with SFM without P/S, and cells were transfected with selected validated siRNA for Egr1 (ID: s4538) or control siRNA (scrambled) (AMBION), to a final concentration of 30 pmol/well (6 well/plate) or 15 pmol/well (12 well/plate) using Lipofectamine RNAiMAX Reagent (Invitrogen) as recommended by the manufacturer. After 6 h, the transfection medium was changed with DMEM/F-12 containing 2,5% FBS-DCC in order to avoid Lipofectamine toxicity. 24h post-transfection cells were exposed to vehicle or G-1 for further 24h and then harvested (RT-PCR and Western blotting) or processed for the viability assay.

### ROS detection

ROS formation inside the cells were quantified using 2,7-dichlorodihydrofluorescein diacetate (H2-DCFDA). The acetate group of H2-DCFDA is removed by the intracellular esterase forming 2′,7′-dichlorofluorescin (DCF). In presence of ROS, non-fluorescent DCF formed is converted into a fluorescent product. The increase in fluorescence intensity is proportional to the oxidation of the fluorescent probe. Cells were seeded at a density of 50 000 cells/well in 24 well/plate and cultured for 48h and then treated with G-1 (1μM) for different times. Moreover, to determine the effects of NAC (5mM) on G-1-induced ROS generation, cells were pretreated 1h before adding G-1 for 24h. After incubation, the cells were loaded with H2-DCFDA (5μM/well) and incubated for 30 min in the dark. The wells were washed with PBS to remove excess of probe. The fluorescence was measured using fluorescent microplate reader (Monochromator based multimode microplate reader, BioTeck Sinergy H1 Instrument) with excitation at 495 nm and emission at 530 nm.

### Protein extraction and Western-blotting

To obtain cytoplasmic and nuclear proteins cells were lifted in ice-cold PBS, transfered to 15 ml tubes, centrifuged for 5 min at 3000 rpm and resuspended in 200 μl of buffer A (10 mM N-2-hydroxyethylpiperazine N′-2-ethanesulfonic acid (HEPES), PH 7.9, 0.1 mM EDTA, 0.1 mM EGTA, 10 mM KCl, 1 mM dithiothreitol and 0.5 mM phenylmethylsulfonyl fluoride). After swelling on ice for 10 min, plasma membranes were disrupted by adding 0.1% Nonidet P-40 and vortexing for 10 sec. Lysates were transferred to 1.5 ml tubes. After centrifugation for 10 min at 10000 rpm at 4°C, supernatant contained cytoplasmic proteins while pellet contained nuclei.

Nuclei were washed twice with ice cold PBS. Nuclei were incubated for 20 min on ice in buffer C (20 mM HEPES PH 7.9, 1 mM EDTA, 1 mMEGTA, 400 mM NaCl, 1 mM dithiothreitol and 1 mM phenylmethylsulfonyl fluoride vortexing every 5 min. Samples were centrifuged at 12,000 rpm for 15 min at 4°C to recover nuclear fraction (supernatant).

Total protein were prepared using RIPA buffer: 50 mM Tris, pH 8.0, 150 mM sodium chloride; 1.0% NP-40, 0.5% sodium deoxycholate, 0.1% SDS (sodium dodecyl sulfate).

Fifty μg of protein were subjected to Western blot analysis. Blots were incubated overnight at 4°C with antibodies against Egr-1, p21^Waf1/Cip1^ and BAX (all from Santa Cruz Biotechnology, Santa Cruz CA, USA). Membranes were incubated with horseradish peroxidase (HRP)-conjugated secondary antibodies (Amersham Pharmacia Biotech, Piscataway, NJ) and immunoreactive bands were visualized with the ECL Western blotting detection system (Amersham). To assure equal loading of proteins, membranes were stripped and incubated overnight with anti-Glyceraldehyde 3-Phosphate DeHydrogenase (GAPDH) or anti-Lamin B (nuclear fraction in Figure 2B) antibodies, (Santa Cruz Biotechnology, Santa Cruz, CA, USA).

### Immunofluorescence and immunohistochemistry

For immunofluorescent analysis (IF), H295R cells were plated on glass coverslips, washed with PBS and fixed with 3.7% formaldehyde in PBS for 20 min at room temperature (RT), followed by permeabilization with 0.2% Triton X-100 in PBS for 3 min at RT. Coverslips were washed with PBS, and nonspecific binding of IgG was blocked with 3% BSA (Sigma) in PBS for 20 min at room temperature. Cells were then incubated overnight in a cold room with an anti-Egr-1 antibody (Santa Cruz Biotechnology,). The following day coverslips were washed three times with PBS, and incubated with fluorescein isothiocyanate-conjugated secondary antibodies (Santa Cruz) for 1h at room temperature. Finally, coverslips were washed three times with PBS and mounted on glass slides with Vectashield mounting medium (Vector Laboratories Inc., CA, USA). Fluorescent images were collected on Olympus fluorescent microscope.

From our previous work we had access to 5 mm thick paraffin-embedded sections of H295R xenograft tumors from mice treated with vehicle and G-1 [16]. Slides were deparaffinized and dehydrated (seven to eight serial sections). Immunohistochemical (IHC) experiments were performed as previously described [16], using Egr-1 primary antibody at 4°C over-night. Then, a biotinylated goat-anti-mouse IgG was applied for 1h at room temperature, to form the avidin biotin-horseradish peroxidase complex (Vector Laboratories, CA, USA). Immunoreactivity was visualized by using diaminobenzidine chromogen (Vector Laboratories). The primary antibody was replaced by normal rabbit serum in negative control sections for both IF and IHC experiments.

### Scoring system

The immunostained slides of tumor samples were evaluated as previously described [43] by using the Allred Score. Briefly, a proportion score was assigned representing the estimated proportion of positively stained tumor cells (0 = none; 1 = 1/100; 2 = 1/100 to <1/10; 3 = 1/10 to <1/3; 4 = 1/3 to 2/3; 5 = >2/3). An intensity score was assigned by the average estimated intensity of staining in positive cells (0 = none; 1 = weak; 2 = moderate; 3 = strong). Proportion score and intensity score were added to obtain a total score that ranged from 0 to 8. A minimum of 100 cells were evaluated in each slide. Six to seven serial sections were scored in a blinded manner for each sample.

### Statistics

All experiments were performed at least three times. Data were expressed as mean values ± standard deviation (SD), statistical significance between control and treated samples was analyzed using GraphPad Prism 5.0 (GraphPad Software, Inc.; La Jolla, CA) software. Control and treated groups were compared using the analysis of variance (ANOVA). A comparison of individual treatments was also performed, using Student’s t test. Significance was defined as p < 0.05.
